# Low Temperature Induced Changes in Citrate Metabolism in Ponkan (*Citrus reticulata* Blanco cv. Ponkan) Fruit during Maturation

**DOI:** 10.1371/journal.pone.0156703

**Published:** 2016-06-01

**Authors:** Qiong Lin, Jing Qian, Chenning Zhao, Dengliang Wang, Chunrong Liu, Zhidong Wang, Chongde Sun, Kunsong Chen

**Affiliations:** 1 Laboratory of Fruit Quality Biology/Zhejiang Provincial Key Laboratory of Horticultural Plant Integrative Biology/The State Agriculture Ministry Laboratory of Horticultural Plant Growth, Development and Quality Improvement, Zhejiang University, Zijingang Campus, Hangzhou, P. R. China; 2 Institute of Food Science and Technology, Chinese Academy of Agricultural Sciences/Key Opening Laboratory of Agricultural Products Processing and Quality Control, Ministry of Agriculture, Beijing, P. R. China; 3 Quzhou Academy of Agricultural Science, Quzhou, P. R. China; Purdue University, UNITED STATES

## Abstract

Citrate is the most important organic acid in citrus fruit, and its concentration in fruit cells is regulated mainly by the balance between synthesis and degradation. Ponkan (*Citrus reticulate* Blanco cv. Ponkan) is one of the major citrus cultivars grew in China, and the fruit are picked before fully mature to avoid bad weather. Greenhouse production is widely used to prolong the maturation period and improve the quality of Ponkan fruit by maintaining adequate temperature and providing protection from adverse weather. In this research, Ponkan fruit cultivated in either a greenhouse or open field were used to investigate differences in the expression of genes related to citrate metabolism during maturation in the two environments. The citrate contents were higher in open field fruit, and were mainly correlated with expressions of *CitPEPCs*, *CitCSs*, *CitAco3* and *CitGAD4*, which were significantly increased. In addition, the impacts of low temperature (LT) and water stress (WS) on citrate metabolism in Ponkan were investigated during fruit maturation. The citrate contents in LT fruit were significantly increased, by between 1.4–1.9 fold, compared to the control; it showed no significant difference in fruit with water stress treatment compared to the control fruit. Furthermore, the expressions of *CitPEPCs*, *CitCSs*, *CitAco3* and *CitGAD4* were significantly increased in response to LT treatment, but showed no significant difference in WS compared to the control fruit. Thus, it can be concluded that low temperature may be the main factor influencing citrate metabolism during maturation in Ponkan fruit.

## Introduction

Organic acid content is one of the most important factors influencing fruit quality. The accumulation of organic acids in plant cells is highly correlated with other metabolism pathways and appears to be under the control of many factors. The synthesis of citrate, a major organic acid in citrus, relies on citrate synthase (CS), which is the key enzyme catalyzing the condensation of acetyl CoA with oxaloacetate (OAA) [1]. OAA is achieved from the carboxylation of phosphoenolpyruvic acid (PEP), which is catalyzed by the phosphoenolpyruvate carboxylase (PEPC) [2]. During development of the fruit, a close correlation was investigated between the content of citrate and PEPC activity in fruits [3]. Aluminum stress induced mitochondria CS activity and promoted the synthesis of citrate in orange roots, and the over-expression of *CjCS* genes improved CS activity and promoted the synthesis of citrate in tobacco [4]. Thus, PEPC and CS are the key enzymes of citrate synthesis pathway in fruit.

The degradation of citrate occurs mainly in the cytosol, catalyzed by the aconitase (Aco), glutamate decarboxylase (GAD) and glutamine synthase (GS) cascade [5]. The reversible isomerization of citrate to iso-citrate is catalyzed by Aco via the intermediate product cis-aconitate. It was suggested that the Aco was closely related to citrate degradation [6]. Over-expression of an *Aco* gene significantly decreased citrate content in tomato [7]. Thus, Aco is considered as the key enzyme of citrate degradation. Citrate is sequentially metabolized to isocitrate, 2-oxoglutarate and glutamate. Thereafter, glutamate is both utilized for glutamine and gamma-aminobutirate (GABA) pathways in citrus [8]. The GABA pathway is the most studied one, where catalyzed by GAD, leads to succinate synthesis [8, 9]. The glutamine pathway may occur during fruit ripening, where GS transforms glutamate into glutamine, which is possibly utilized for thiamine biosynthesis [8].

The accumulation and metabolism of organic acids can be regulated by many environmental factors. As shown in the grape berry and in banana, the higher temperature during fruit growth or storage, the lower concentrations of malate and citrate [10, 11]. Yun et al. showed that low temperature inhibited primary metabolism, secondary metabolism and the transportation of metabolites during storage of pummelo fruit [12]. The above phenomenons probably result from the impact of temperature on modifying activity of enzymes related to glycolysis and the tricarboxylic acid (TCA) cycle [13]. The process-based simulation model (PBSM) of fruit indicated that the rate of citrate synthesis (or degradation) was expressed as a function of temperature, fruit mesocarp weight and respiration [14]. Further model indicated that fruit acidity was affected by temperature differently among the fruit cultivars or species [15].

Water is another key factor affecting organic acid metabolism. According to previous reports, sufficient water supply decreased the organic acid content in ripening fruits [16, 17]. However, no obvious change was investigated in the seasonal patterns of the organic acids accumulation [18, 19]. It was suggested that water stress influencing organic acid content simply through a dilution/dehydration effect [20]. The osmotic adjustment under water stress, mainly accumulated sugars and organic acids to regulate their osmotic potential and prevent a drop in cell turgor pressure, may be another key factor affecting the acidity of fruit cells [21]. Previous reports also indicated the accumulation of organic acids in the leaves and xylem fluid of plants [21, 22], there is a possibility that water stress may increase the import of organic acids to the fruit.

Ponkan (*Citrus reticulate* Blanco cv. Ponkan) is a major citrus cultivar grew in China, and fruit are picked before fully mature to avoid bad weather in the winter, thus, leading to high acidity of fruit. To prolong the maturation period of Ponkan fruit, the greenhouses are widely used for production, which can maintain adequate temperature and promote fruit quality. In this research, the difference in citrate synthesis and degradation in Ponkan fruit cultivated in a greenhouse and open field during delayed harvest was studied, and the effects of environmental factors, mainly low temperature and water stress, on citrate metabolism were analyzed.

## Materials and Methods

### Plant Material and Treatments

Ponkan (*C*. *reticulata* Blanco cv. Ponkan) fruit cultivated both in the field and in pots in a glasshouse were used as materials in present study.

Developmental series of fruit cultivated in the field were collected from orchards belong to Quzhou Academy of Agricultural Science located in Quzhou, Zhejiang, China. This is permitted by Chunrong Liu, who is the responsible person of the institute (Chunrong Liu, E-mail:qzlcr@aliyun.com). Twelve fruit were selected at each sampling point at 60 (S1), 90 (S2), 120 (S3), 150 (S4), 180 (S5), 195 (S6), 210 (S7) and 225 (S8) days after flowering. The S5 stage was the commercial harvest time. In this study, the fruits were allowed to remain on the trees for both the greenhouse and open field cultivation treatments from S5 to S8 stages.

Ponkan plants in pots were grown under regular cultivation during the early developmental stages, and were transferred to climate chambers for the temperature and water treatment at 180 day after flowering (Commercial harvest stage). The Ponkan plants were subjected to the following treatment: (1) Control (CK): 25°C for 16 hours followed by 20°C for 8 hours; regular watering. (2) Low temperature (LT): Natural low temperature in winter; regular watering. (3) Water stress (WS): 25°C for 16 hours followed by 20°C for 8 hours; maximum water holding capacity was maintained at 20%. (4) Low temperature plus water stress (LT+WS): Natural low temperature in winter; maximum water holding capacity was maintained at 20%. Twelve fruit were picked at 0, 30 and 60 days after each treatment.

### Total Soluble Solids (TSS) and Titratable Acids (TA) Analysis

TSS was measured using a digital hand-held refractometer (PR101-α, Atago, Japan). Three drops of juice from one segment were measured, and the procedure was repeated twice per fruit with nine single fruit replicates. TA of juice sacs was titrated with 0.1 N NaOH to the end point at pH 8.2. All the above methods were described in detail by Chen et al. [23].

### Organic Acids Analysis

The citrate content was measured according to method in previous reports, which has been described in detail [24–26]. Mixed samples of 0.1 g were extracted with 1 ml of methanol at 70°C for 15 min, and centrifuged at 10,000 g. The dried residue was incubated with pyridine methoxyamine hydrochloride and Bis (trimethylsilyl) trifluoroacetamide (1% trimethylchlorosilane). In this analysis, 10 μl ribitol (0.2 mg/ml) was added to each sample as an internal standard. The sample was injected into a gas chromatography-mass spectrometer (GC-MS) fitted with a fused-silica capillary column (30 m × 0.25 mm i.d., 0.25 μm DB-5 MS stationary phase). The injector temperature was 250°C and the helium carrier gas had a flow rate of 1.0 ml/min. The column temperature was held at 100°C for 1 min, increased to 185°C with a temperature gradient of 3°C/min, increased to 250°C at 15°C/min and then held for 2 min.

### RNA Extraction and cDNA Synthesis

Total RNA was prepared according to our previously reported method [27]. Contaminating genomic DNA was digested by TURBO DNA free kit (Ambion). First strand cDNA was synthesized from 1.0 μg DNA-free RNA, using iScriptTM cDNA Synthesis Kit (Bio-Rad). Three biological replicates of each sample were used for RNA extraction and subsequent cDNA synthesis.

### Oligonucleotide Primers and Quantitative Real-time PCR (qRT-PCR)

The primers for qRT-PCR analysis were designed with the online software primer 3 (http://frodo.wi.mit.edu/primer3/). The specificity of primers was determined by melting curves and PCR products re-sequencing. The sequences of oligonucleotide primers are described in Table 1.

**Table 1:** Primers used for quantitative real-time PCR analysis.

Gene	Forward primer (5´ to 3´)	Reverse primer (5´ to 3´)
*CitPEPC1*	GCCCGGGAATATTTGTACG	AAGGCTCAAGGCCACTTTTT
*CitPEPC2*	TCGTGTTGCTTTCACCAAAA	AATGCTTTCTTTGCAGCGTA
*CitPEPC4*	CAATTCGCTAGCTGGTTTAACA	ACCATCAAGCCGCAGTTCTA
*CitCS1*	CGAGGCCATTTGATTACTGC	TGTTGGCCATTTTGTAACCA
*CitCS2*	GGCCTCTGGTTTTCTGTTTG	ACAATACCACGCAGGGAAAG
*CitAco1*	CCAACTGGTGCTCTTCAACC	TGTCTAGGGCGTGTCCTATTG
*CitAco2*	CTAGGCGGACTTGCTTCATC	CCTTTTGAATTGTTCCCAGAA
*CitAco3*	GCATGAGGCATGAGGATTC	TTGGCCAAAAGAAAAATGAA
*CitGAD4*	CGTCTCCGAAAGGAAAGCTA	AAATAATCAAAATCGTCAACATGC
*CitGAD5*	GGTTCCAAACATAAGACGAGACA	CACTTCATAAGCGTGCAAGAA
*CitGS2*	TGAGCATCGATGACGAAGAA	TGGCAAGGAACAAGTTCAAA
*CitActin*	CATCCCTCAGCACCTTCC	CCAACCTTAGCACTTCTCC

The qRT-PCR was carried out with Ssofast EvaGreen Supermix kit (Bio-Rad) and a CFX96 instrument (Bio-Rad) for gene expression studies according to the method described in detail by Yin et al. [27]. The PCR reaction mixture (20 μl total volume) comprised 10 μl 2 × real-time PCR mix (Bio-Rad), 1 μl of each primer (10 μM), 2 μl diluted cDNA, and 6 μl DEPC H_2_O. The PCR program was initiated for 30 s at 95°C, followed by 44 cycles of 95°C for 5 s, 60°C for 5 s, and completed with a melting curve analysis program. The efficiency of amplification of different primers was rather similar and close to 100%, and was assumed to be 100% in this study. The actin gene was used as an internal control [23], and was considered to be stable in the conditions of the present study (data not shown). The relative expression of genes in Ponkan fruit was calculated by △Ct method.

### Statistical Analysis

Least significant differences (LSD) at the 0.05 level was calculated by DPS 7.05 (Zhejiang University, Hangzhou, China). Figures were drawn using Origin 8.0 (Microcal Software Inc., Northampton, MA, USA).

## Results

### TSS and TA Contents Variation in Ponkan Fruit during Development and Delayed Harvest

**Fig 1:**
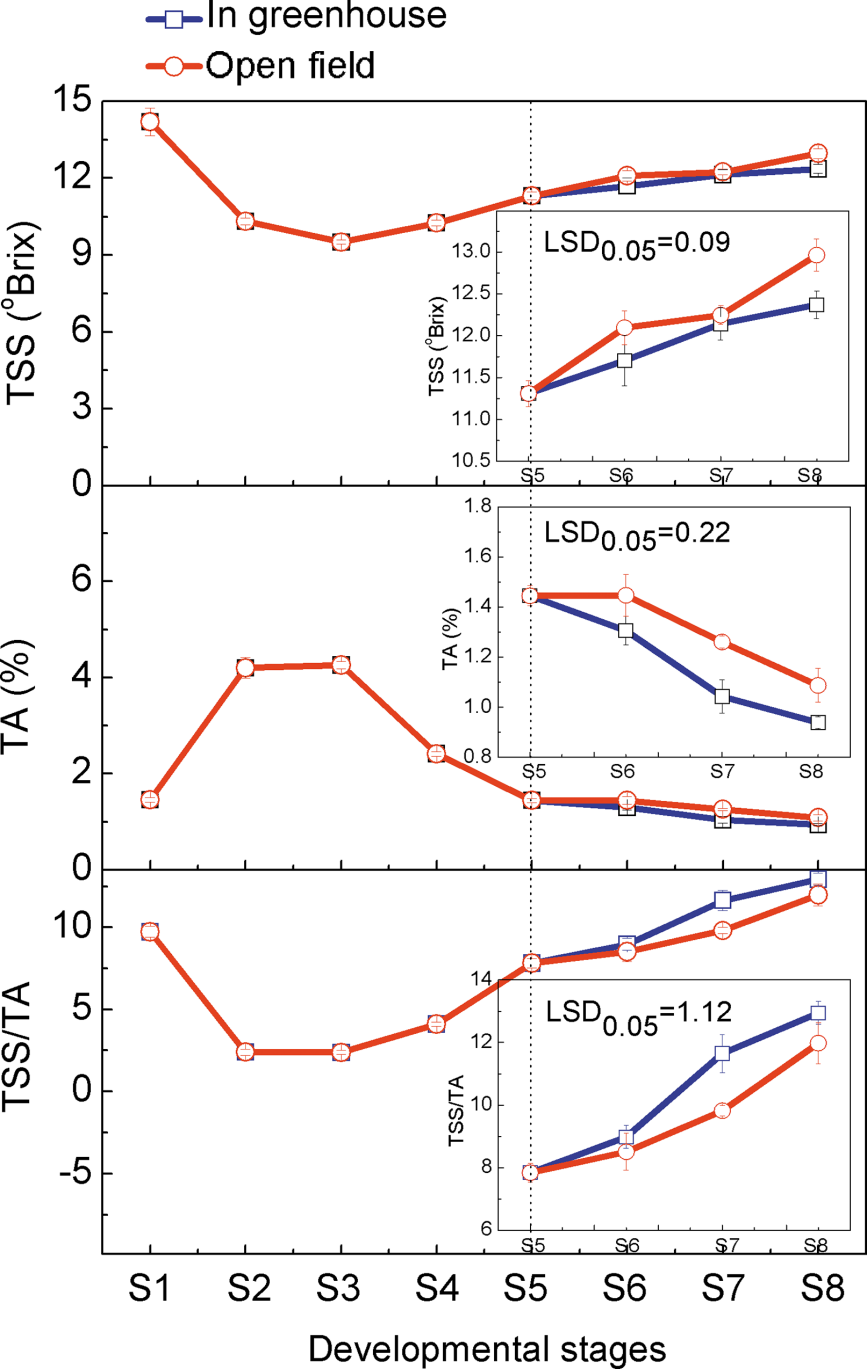
TSS and TA contents in Ponkan fruit during development and delayed harvest. The error bars represent the standard errors. LSDs represent least significant differences at the 0.05 level, and were calculated with data from stages S5 to S8.

During different stages of Ponkan fruit development, the TSS contents were relatively stable from S2 to S4 stages and increased rapidly from stage S5 to S8. During the delayed harvest, the TSS contents in greenhouse fruit were lower than in open field fruit, by 1%-3%. The TA contents increased from S1 to S3, and then decreased rapidly from S4 to S8. During the delayed harvest stages, the TA contents in greenhouse fruit were lower than in open field fruit, by 10%-20%. From stage S6 to S8, the ratio of TSS to TA content in greenhouse fruit was higher than in open field fruit, by 6%, 19% and 8%, respectively, which indicated the better flavor of the greenhouse fruit (Fig 1).

### Organic Acids Contents Variation in Ponkan Fruit during Development and Delayed Harvest

**Fig 2:**
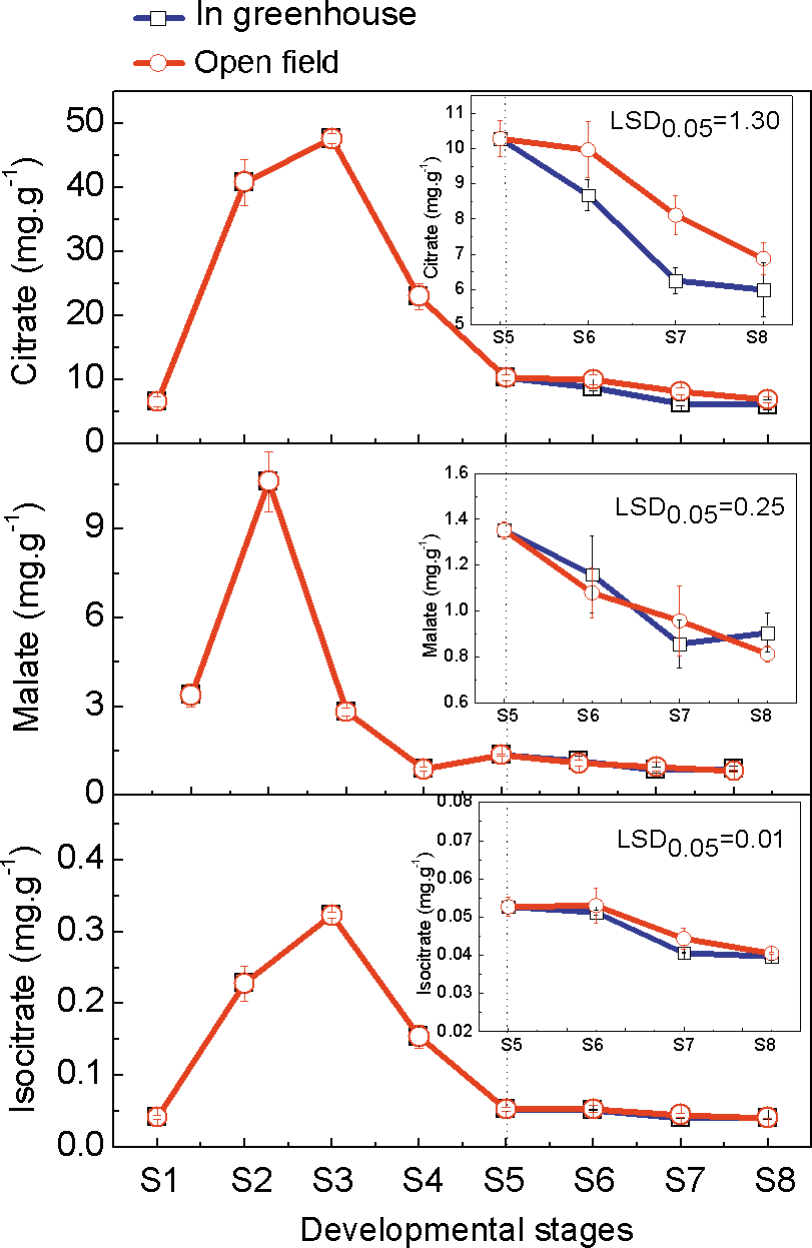
Contents of organic acids in Ponkan fruit during development and delayed harvest. The error bars represent the standard errors. LSDs represent least significant differences at the 0.05 level, and were calculated with data from stages S5 to S8.

The contents of three main organic acids in Ponkan fruit at different developmental stages were analyzed. Citrate content accounted for 65%-95% of the total acids, and was significantly higher than malate and isocitrate during Ponkan fruit development stages. All the three organic acids increased during the early developmental stages, but decreased at later stages. The highest citrate and isocitrate contents were observed at the S3 stage, while S2 was the highest stage of malate. During the delayed harvest stages, citrate contents in greenhouse fruit were significantly decreased, by 15%, 29% and 15%, respectively, compared with that in open field fruit. However, there was no significant difference in malate and isocitrate contents between greenhouse fruit and open field fruit (Fig 2).

### Expressions of Citrate Synthesis Related Genes in Ponkan Fruit during Delayed Harvest

**Fig 3:**
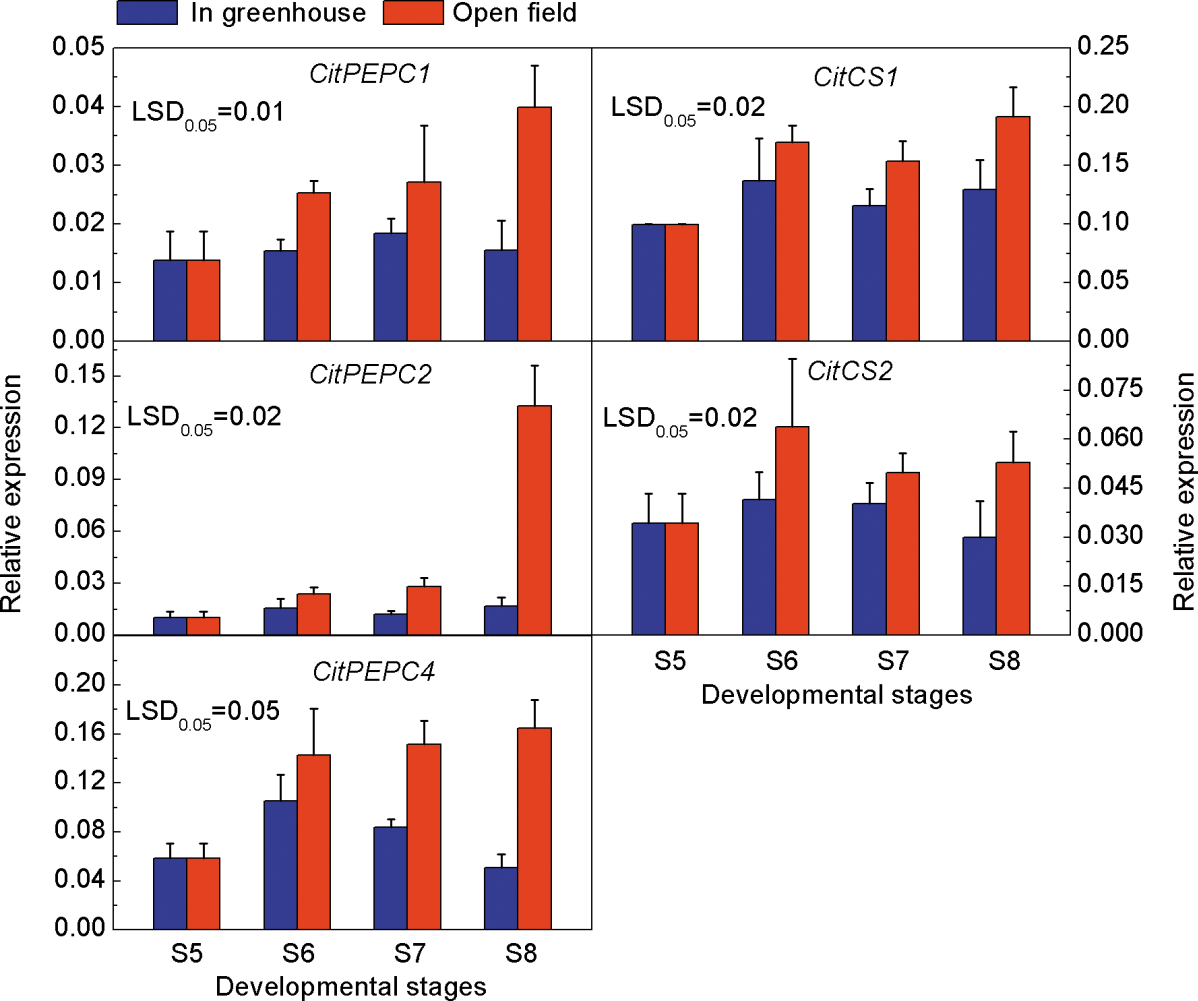
Expressions of genes related to citrate synthesis in Ponkan fruit cultivated in a greenhouse and open field. The error bars represent the standard errors. LSDs represent least significant differences at the 0.05 level.

PEPC and CS are the key enzymes in the citrate synthesis pathway. During the delayed harvest stages, the expressions of three *CitPEPC* members in open field fruit were significantly higher than that in greenhouse fruit and the expressions of *CitPEPC1*, *CitPEPC2* and *CitPEPC3* at stage S8 were up-regulated, by 2.6, 7.9 and 3.3 fold, respectively. The expressions of *CitCS1* and *CitCS2* were also significantly up-regulated in open field fruit compared to greenhouse fruit during delayed harvest (Fig 3).

### Expressions of Genes related to Citrate Degradation in Ponkan Fruit during Delayed Harvest

**Fig 4:**
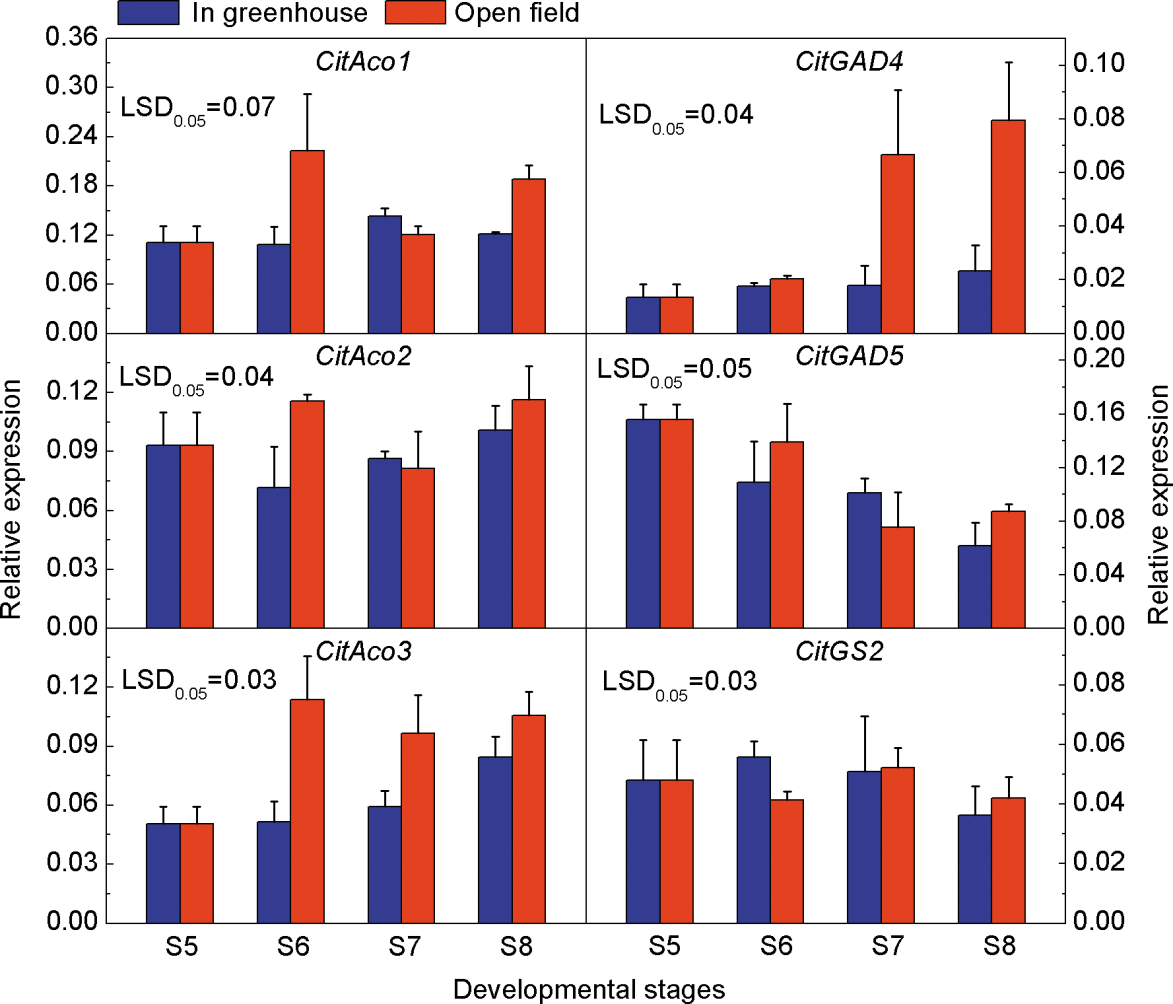
Expressions of genes related to citrate degradation in Ponkan fruit cultivated in greenhouse and open field. The error bars represent the standard errors. LSDs represent least significant differences at the 0.05 level.

Aco is the first key enzyme of the citrate degradation pathway, which catalyzes conversion of citrate to isocitrate. At stage S6, the expressions of *CitAco1* and *CitAco2* were significantly higher in open field fruit than that in greenhouse fruit, but showed no significant differences thereafter. The expression of *CitAco3* in greenhouse increased during the delayed harvest stages, and was significantly higher in open field fruit than in greenhouse fruit at stages S6 and S7, by 2.2 and 1.7 fold, respectively. GAD and GS are the key enzymes of the GABA pathway and glutamine pathway. The expression of *CitGAD4* was relatively stable during the delay harvest stages, and was significantly higher in open field fruit than that in greenhouse fruit at stages S7 and S8, by 3.7 and 3.4 folds, respectively. The expression of *CitGAD5* decreased during the delayed harvest stages, but there was no significant difference between the treatments. *CitGS2* showed irregular expression, and no significant difference was found between the treatments (Fig 4).

### Effects of LT and WS on Citrate Accumulation in Ponkan Fruit during Delayed Harvest

**Fig 5:**
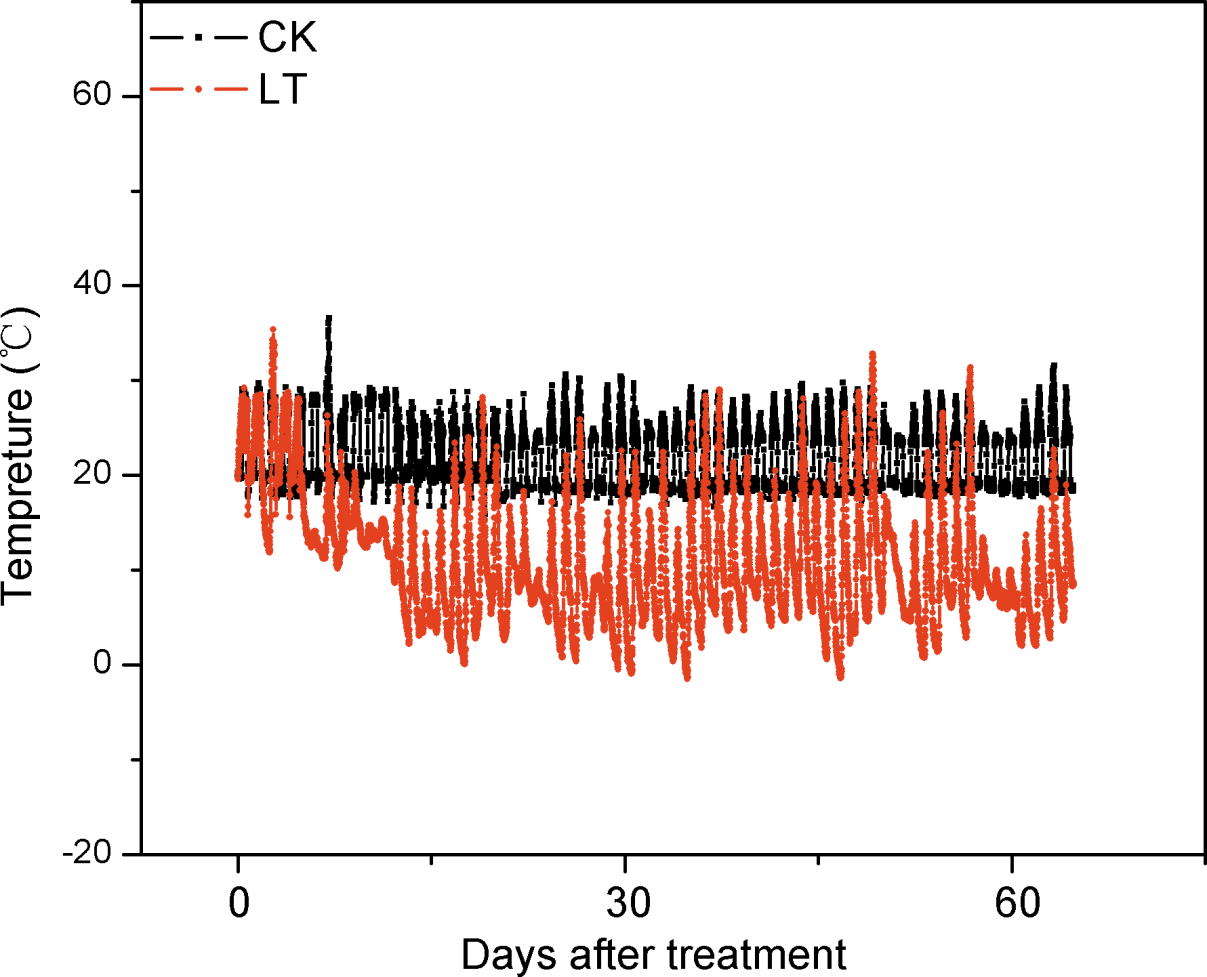
Real-time temperature during treatment of Ponkan plants.

**Fig 6:**
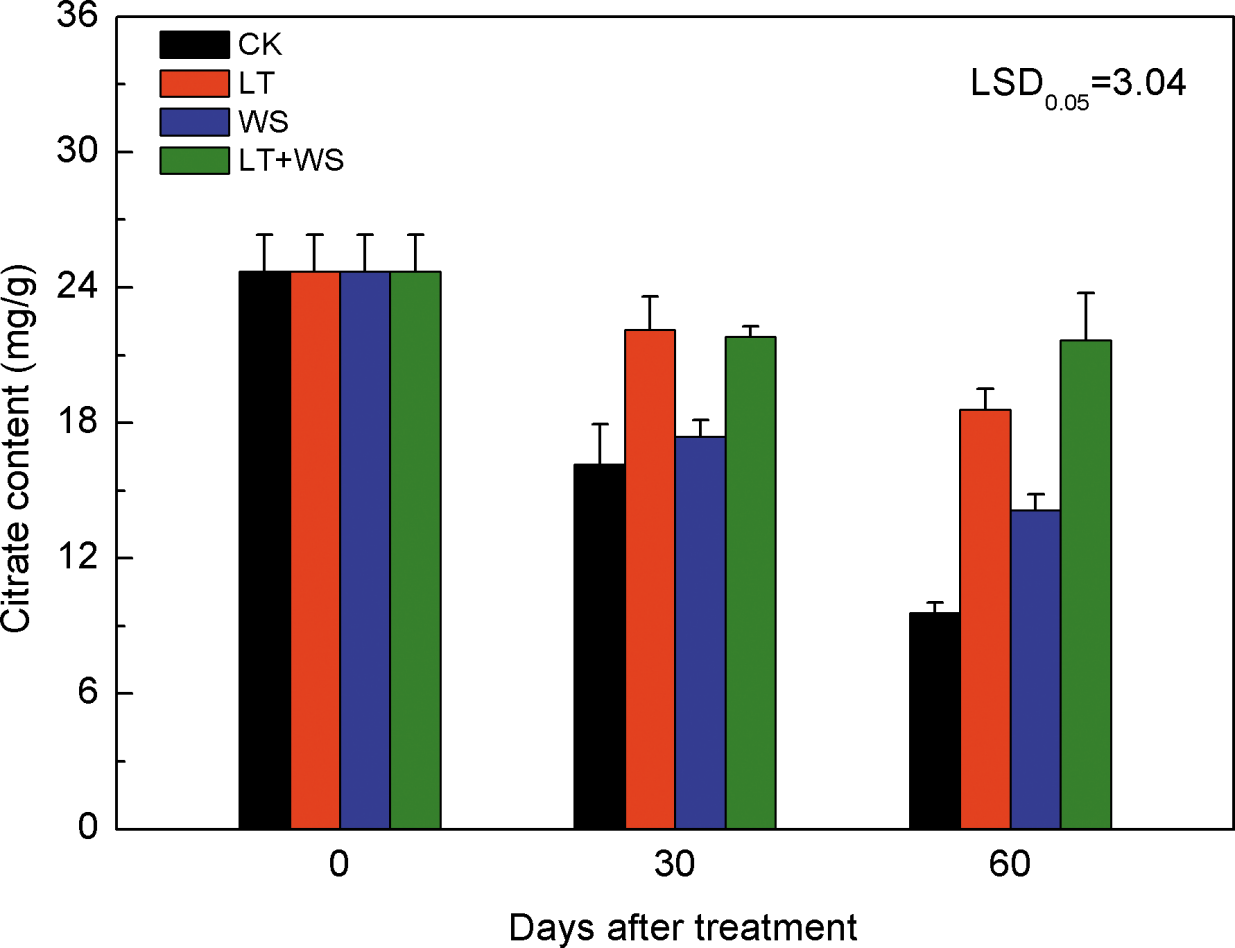
Citrate contents of Ponkan fruit during low temperature and water stress treatments. CK: the control; LT: low temperature; WS: water stress; LT+WS: low temperature plus water stress. The error bars represent the standard errors. LSDs represent least significant differences at the 0.05 level.

The real-time temperature during treatment of Ponkan plant is shown in Fig 5. The citrate content in Ponkan fruit showed a downward trend in all the treatments during the delayed harvest, and decreased by 61%, 25%, 43% and 12% at 60 days after treatment in CK, LT, WS and LT+WS, respectively. Compared to the CK fruit, the citrate content increased significantly in LT treated fruit at 30 and 60 days after treatment. No significant difference in citrate content was found in WS treated fruit at 30 days after treatment, but it increased significantly at 60 days after treatment compared to the CK fruit. After 30 days treatment, the citrate content in LT+WS treated fruit increased significantly compared to the CK and WS treated fruit, but no significant difference were observed compared to the LT treated fruit. After 60 days treatment, the citrate content in LT+WS treated fruit increased 1.9, 1.5 and 2.3 fold, respectively, compared to CK, WS and LT treated fruit (Fig 6).

### Effects of LT and WS on Citrate Synthesis in Ponkan Fruit during Delayed Harvest

**Fig 7:**
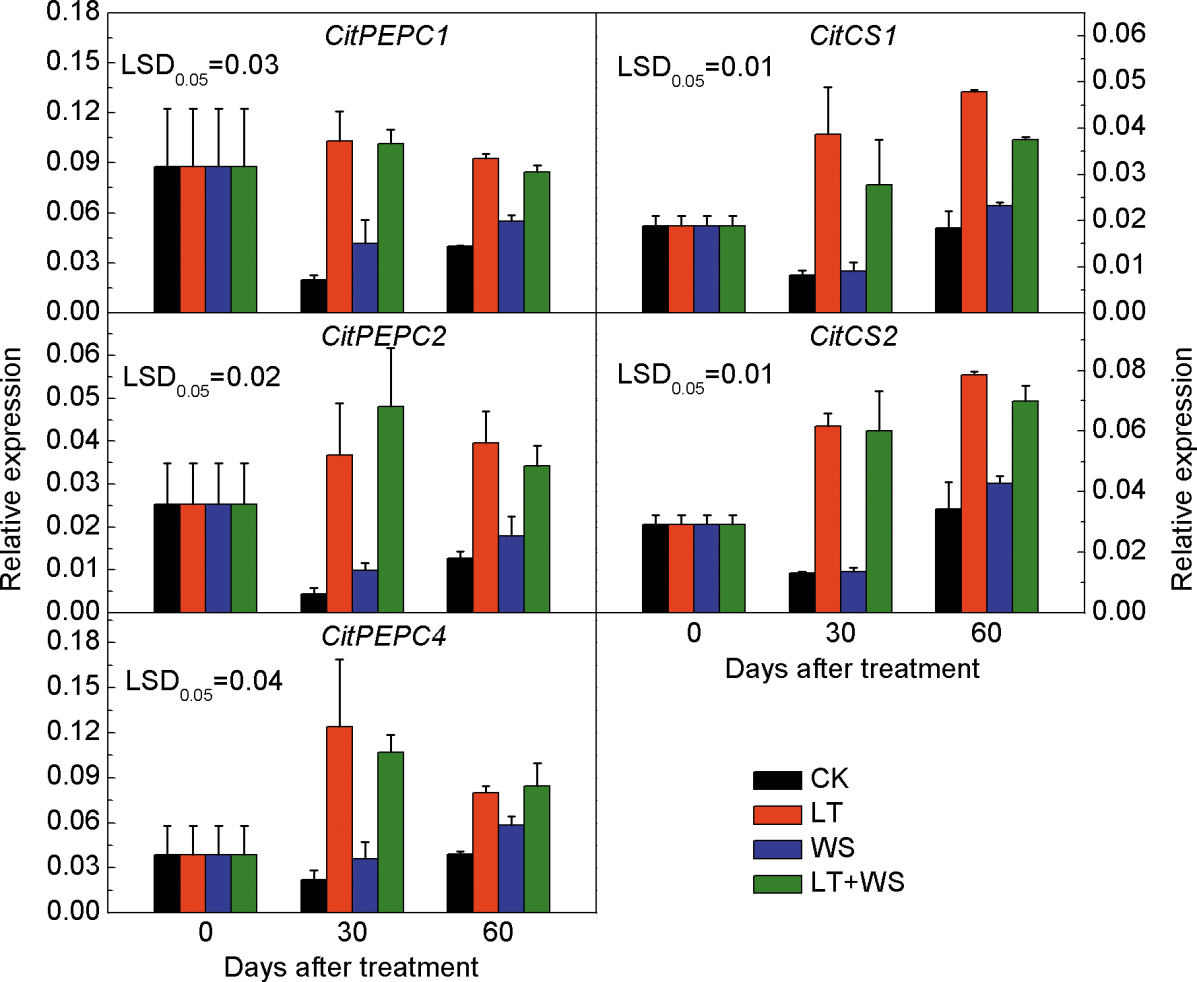
Expressions of genes related to citrate synthesis in Ponkan fruit during low temperature and water stress treatments. The error bars represent the standard errors. LSDs represent least significant differences at the 0.05 level. CK: the control; LT: low temperature; WS: water stress; LT+WS: low temperature plus water stress.

The expressions of three *CitPEPC* genes showed similar trends during treatment of Ponkan fruit. Compared to the CK fruit, expressions of *CitPEPC1*, *CitPEPC2* and *CitPEPC3* in LT and LT+WS treated fruit increased significantly at 30 and 60 days after treatment, but no significant difference was observed in WS treated fruit. The expression of these three genes in LT+WS treated fruit increased significantly compared to WS treated fruit, but no significant difference was found when compared to the LT treated fruit. Compared to the CK fruit, the expressions of *CitCS1* and *CitCS2* in LT and LT+WS treated fruit increased significantly at 30 and 60 days after treatment, but no significant difference was found in WS treated fruit (Fig 7). The results showed that the expressions of *CitPEPCs* and *CitCSs* were induced in response to low temperature in Ponkan fruit during delayed harvest.

### Effects of LT and WS on Citrate Degradation in Ponkan Fruit during Delayed Harvest

**Fig 8:**
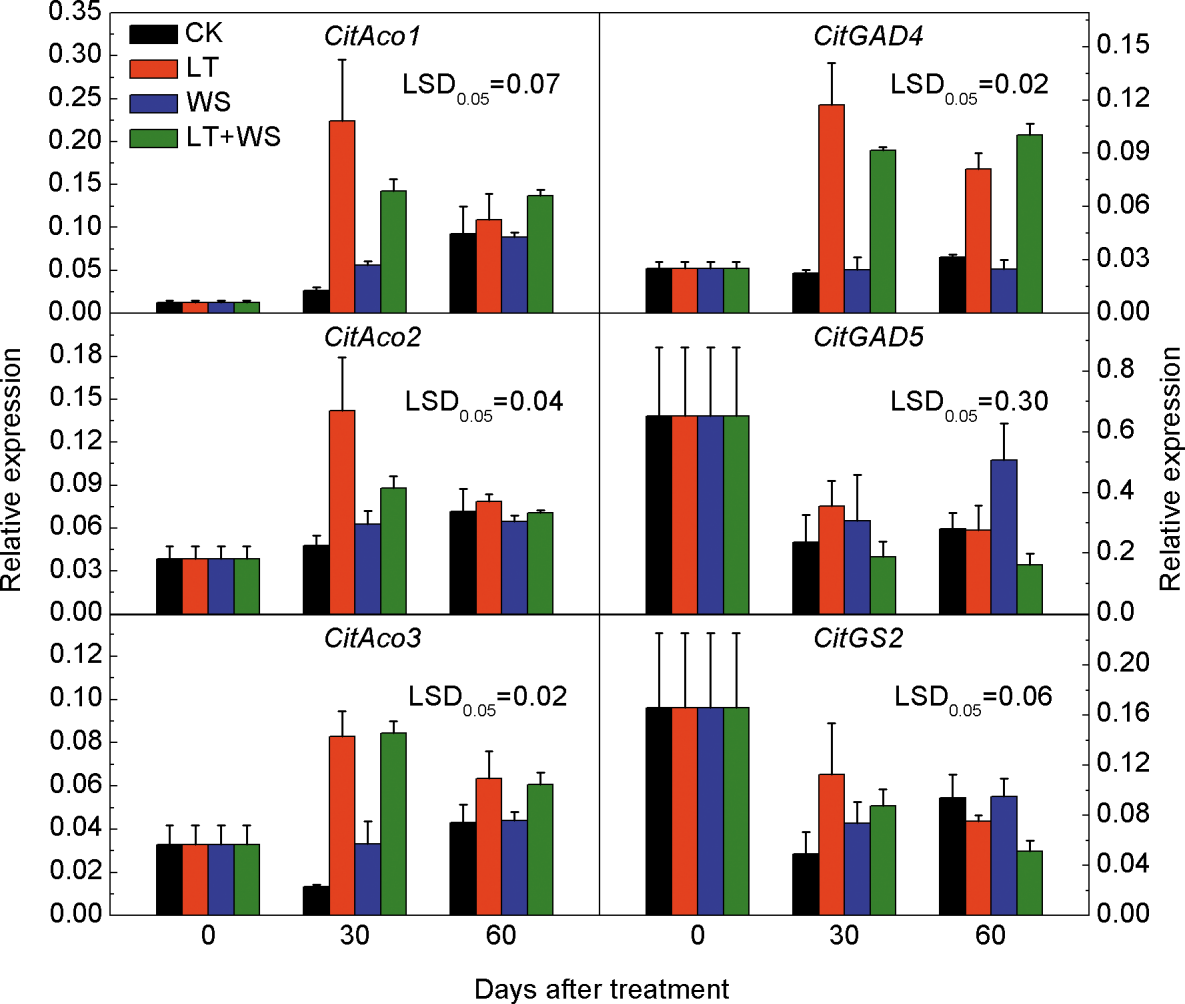
Expressions of genes related to citrate degradation in Ponkan fruit during low temperature and water stress treatments. The error bars represent the standard errors. LSDs represent least significant differences at the 0.05 level. CK: the control; LT: low temperature; WS: water stress; LT+WS: low temperature plus water stress.

Compared to the CK fruit, the expressions of *CitAco1* and *CitAco2* in LT and LT+WS treated fruit increased significantly at 30 days after treatment, by 8.3 and 5.3 fold, but no significant difference was found at 60 days after treatment. The expressions of *CitAco1* and *CitAco2* in WS treated fruit were not significantly different from CK fruit during the treatment. At 30 days after treatment, the expression of *CitAco3* in both LT and LT+WS treated fruit increased significantly; At 60 days after treatment, the *CitAco3* expression in LT treated fruit was significantly different from CK fruit, but no significant difference was found in LT+WS treated fruit compared to the CK fruit and similarly there was no significant difference between the WS and CK treated fruit during the treatment. Expression of *CitGAD4* in LT and LT+WS treated fruit increased significantly, however, compared to CK fruit, no significant difference was found between the WS and CK treatment. The expressions of *CitGAD5* and *CitGS2* showed a downward trend during the treatment, but no significant difference was found between treatments (Fig 8). The results indicated that *CitAco3* and *CitGAD4* were significantly induced by low temperature.

## Discussion

Ponkan is a major mandarin citrus grew in China, and the annual production is about 4 million tons, of which 0.5 million tons are from Quzhou City, Zhejiang Province [28]. Ponkan fruit produced in Quzhou has a strong flavor that favors marketing. However, the fruit must be harvested early before they are fully matured to avoid bad weather, which leads to high acidity of Ponkan fruit. To improve fruit quality, greenhouses are widely used in Ponkan production, which can maintain adequate temperature and provide a relatively stable environment of Ponkan fruit development during the later ripening stage [29]. Our results showed that the greenhouse cultivation can decrease citrate content and improve the solids to acids ratio, thus, improving the fruit flavor during maturation of Ponkan fruit.

Organic acids play important roles in plant responses to various stresses. For example, aluminum stress induced citrate accumulation in rye, corn and barley, indicating a close correlation between citrate content and aluminium stress in plants [30–32]; drought promoted the accumulation of citrate in the leaves of cotton [33]; dry soil increased acidity of citrus fruit during later stages of maturation [34]; acidity was increased after frost injury in orange [35]. It was suggested that organic acid accumulation may play a role in improving plant tolerance to stress. Our results showed that citrate was accumulated in Ponkan fruit grew in open fields, indicating that it may play a role in the process of resisting adverse environment, which were not suitable for fruit maturation.

The main factors influencing open field fruit quality in winter is the low temperature and physiological drought. Temperature is the key factor regulating citrate accumulation and metabolism. Gong et al. found that the higher average temperature, annual sunshine hours and accumulated temperature, the lower organic acid content in fruit [36]; it has also been reported that a large amount of citrate was accumulated in citrus fruit after frost injury [35]. During the later harvest stages of Ponkan fruit, the average daily temperature is decreasing rapidly and unstable, the fruit may be at risk from extreme low temperature, thus, this may be the main reason for citrate accumulation in open field fruit. Furthermore, such a response has been verified in potted Ponkan fruit after low temperature processing. In addition, a lack of water absorption ability in root of open field citrus fruit could also cause physiological drought. Early research suggests that water deficiency has no effect on the seasonal change of malate and citrate content [15] and water treatment does not affect the citrate content of mango fruit [37]; however, it has also been suggested that the citrate content increased in satsuma mandarin under water stress. Our results showed no significant difference in citrate content of fruit under water stress and control after 30 days treatment, indicating perhaps that the influence of water stress on citrate content is a long-term effect. Therefore, low temperature seems to be the main factor influencing citrate accumulation in open field fruit during maturation.

GABA is correlated to both biotic and abiotic stresses. The GABA shunt was reported to be associated with regulation of cytosolic pH, carbon fluxes into the TCA cycle, nitrogen metabolism, deterrence of insects, and protection against oxidative stress, osmoregulation and signaling [38]. The content of GABA in vivo is generally low in plants grown in normal condition, while GABA increased rapidly and reached to a high level under stress [39]. The change in GABA metabolism may be associated with the carbon/nitrogen balance in plants or over-expression of genes associated with GABA synthesis under stress [38]. The GABA pathway also participates in citrate metabolism during fruit ripening or under hot air treatment [9, 25]. Our results suggested the GABA pathway is also activated by low temperature in Ponkan fruit during maturation.

In summary, citrate accumulated in Ponkan fruit during maturation under unsuitable growth environments, which was mainly regulated by low temperature. The large amount of citrate may promote the synthesis of GABA, but the specific mechanism needs further research.
